# The effect of the Er,Cr:YSGG laser combined casein phosphopeptide amorphous calcium phosphate for enamel remineralisation: a systematic review and meta-analysis of in vitro studies

**DOI:** 10.1007/s10103-023-03864-5

**Published:** 2023-09-05

**Authors:** Lin Cheng, Rui Yuan, Hao Fan, Minmin Si, Zhaonan Hao, Zhiyuan Feng

**Affiliations:** 1 Shanxi Bethune Hospital, Shanxi Academy of Medical Sciences, Tongji Shanxi Hospital, Third Hospital of Shanxi Medical University, Taiyuan, China; 2 Tongji Hospital, Tongji Medical College, Huazhong University of Science and Technology, Wuhan, China; 3 School and Hospital of Stomatology, Shanxi Province Key Laboratory of Oral Diseases Prevention and New Materials, Shanxi Medical University, Taiyuan, China; 4 Department of Orthodontics, The Fifth Clinical Medical College of Shanxi Medical University, Taiyuan, China

**Keywords:** Casein phosphopeptide-amorphous calcium phosphate, Er,Cr:YSGG laser, Remineralisation, Meta-analysis

## Abstract

The purpose of this systematic review and meta-analysis of in vitro studies was to evaluate the effect of the 2780 nm Er,Cr:YSGG laser combined with casein phosphopeptide-amorphous calcium phosphate (CPP-ACP) for enamel remineralisation. The electronic PubMed, Cochrane Library, Web of Science, and EMBASE databases were searched, with no language or date restrictions, up to January 2023. Two reviewers independently performed research information extraction and quality assessment. Continuous variables were analysed by standard mean difference (SMD) with a 95% confidence interval (CI). The statistical analyses were conducted using Review Manager (Version 5.4; Rev Man) and Cochrane Collaboration (2020). Finally, four trials were included for meta-analysis. According to the comprehensive results, the effect of the Er,Cr:YSGG laser combined with CPP-ACP on enamel remineralisation was significantly better than that of CPP-ACP alone: surface microhardness (SMD =  − 1.83, 95% CI: [− 2.98, − 0.69], *P* = 0.002); lesion depth (SMD = 6.63, 95% CI: [4.98, 8.28], *P* < 0.001). Under the limitations of this meta-analysis, the results show that the Er,Cr:YSGG laser combined with CPP-ACP has a better effect on enamel remineralisation than CPP-ACP alone. The combination of the Er,Cr:YSGG laser and CPP-ACP may be a feasible method to prevent and treat enamel demineralisation.

## Introduction

Malocclusion can be effectively treated with fixed appliances, but the oral hygiene condition of patients under orthodontic treatment is difficult to maintain due to the presence of brackets. Plaque biofilms are more likely to form and produce acid, which dissolves the main component of enamel, hydroxyapatite, resulting in enamel demineralisation [1]. Due to the loss of minerals in the enamel, the clinical manifestations are chalky and opaque plaque, and these lesions are often termed white spot lesions (WSLs) [2], marking the formation of early enamel caries [3]. As the demineralisation process continuous, tooth cavities eventually form [4]. The formation of WSLs not only affects the aesthetics of patients but also seriously damages the dental tissue. A meta-analysis reported a pooled incidence of 45.8% and a pooled prevalence rate of 68.4% of WSLs after fixed orthodontic treatment [5], which demands great attention from both patients and doctors and measures should be taken to prevent the occurrence of WSLs.

Preventing the formation of WSLs can be achieved by inhibiting demineralisation and promoting the remineralisation of early lesions [6]. Remineralisation is a repair process that restores minerals. Studies have shown that mineralising agents containing high calcium and phosphate ions can reverse WSLs [7]. However, due to the inherent insolubility of calcium phosphate, soluble calcium and phosphate ions can only be used at low concentrations. As a result, soluble calcium and phosphate ions do not create an effective concentration gradient to drive diffusion into the subsurface enamel [8].

To overcome this difficulty, a new agent has been developed that uses phosphopeptides from the milk protein casein to stabilise calcium and phosphate ions on the tooth surface and promote enamel remineralisation [8]. This bioactive agent is known as casein phosphopeptide-amorphous calcium phosphate (CPP-ACP) and has recently been introduced [9, 10]. Casein phosphopeptide (CPP) binds calcium and phosphate through phosphoserines in its chemical composition and forms small calcium phosphate clusters (ACP), and the highly insoluble calcium and phosphate are dissolved in the presence of CPP [11]. When CPP-ACP was applied to the tooth surface, the nanocomplex diffused into the porosities of an enamel subsurface lesion and diffused down the concentration gradient into the subsurface lesion site [12]. CPP-ACP releases the weakly bound calcium and phosphate ions, which then deposit into crystal pores, enhancing remineralisation [13].

To further enhance the effect of CPP-ACP on enamel remineralisation, some scholars have combined CPP-ACP with lasers [14, 15]. Studies have shown that laser irradiation can make enamel more resistant to acid dissolution and reduced subsurface demineralisation [16]. Nevertheless, the actual mechanisms remain unclear. The possible mechanisms include the following: (1) decreasing enamel permeability by melting enamel crystallisation and recrystallisation; (2) decreasing enamel solubility by the formation of less soluble complexes such as tetracalcium diphosphate monoxide; and (3) decreasing enamel solubility by ultrastructure changes, such as reducing the water and carbonate content of the enamel, increasing its hydroxyl ion content and promoting the formation of pyrophosphate [17].

Various lasers have been studied to prevent enamel demineralisation, including argon lasers, CO_2_ lasers, Nd-YAG lasers, Er: YAG lasers, and Er,Cr:YSGG lasers [18]. Among them, the Er,Cr:YSGG laser emits light at 2.78 μm, and the wavelength is highly absorbed by water and hydroxyl ion in the hydroxyapatite [19]. Therefore, this type of laser has the potential to prevent mineral loss by changing the chemical composition and morphological structure of tooth enamel without generating excessive heat, which reduces the damage to the enamel, showing the advantages of Er,Cr:YSGG laser compared with other wavelengths of laser [20].

However, the cumulative effect of lasers and CPP-ACP on enamel remains to be discussed. Some researchers have suggested that the combination does not promote the effect of CPP-ACP on increasing enamel surface hardness [15, 21]. Also, a meta-analysis has not been conducted to evaluate whether the combination of Er,Cr:YSGG laser and CPP-ACP can produce a synergistic effect on enamel remineralisation. Hence, the present study aimed to conduct a systematic review and meta-analysis of in vitro studies comparing the effect of enamel remineralisation with topical CPP-ACP and the Er,Cr:YSGG laser irradiation combination therapy versus CPP-ACP alone.

## Materials and methods

### Protocol and registration

**Fig. 1:**
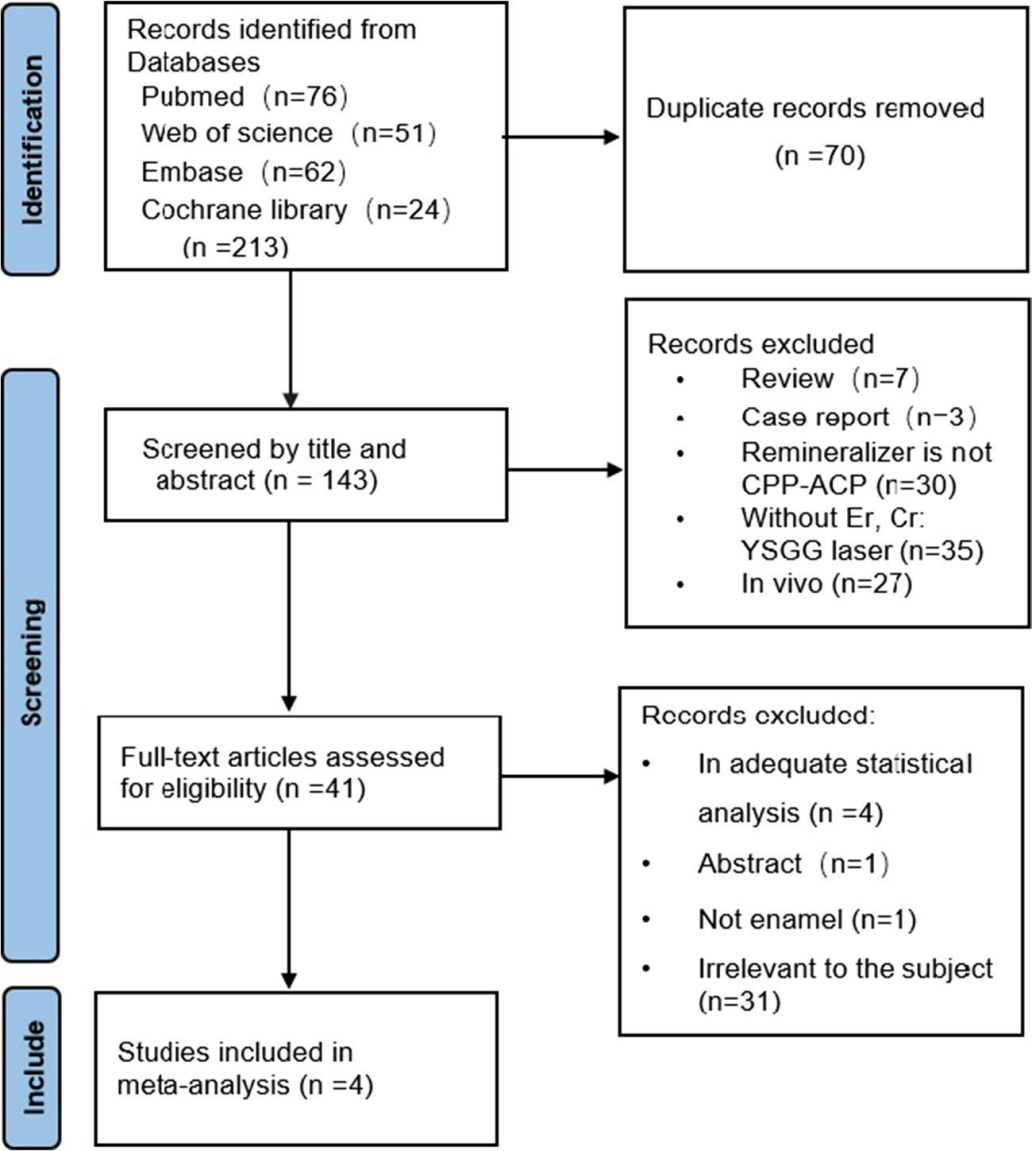
Prisma flow diagram

This systematic review was conducted and reported according to the Cochrane Handbook for Systematic Reviews of Interventions [22] and the PRISMA statement [23]. This study was registered with PROSPERO (CRD42022312818). The PRISMA flow diagram is shown in Fig. 1.

### Search strategy

To find published research reports, the following electronic databases were searched: PubMed, Cochrane Library, Web of Science, and EMBASE. Table 1 summarises the search terms and search strategies. Two authors independently used these search strategies to review the title and abstract, with no language and date restrictions, up to January 2023.

**Table 1:** Search strategy in the four databases

Database	Search strategy
PubMed https://pubmed.ncbi.nlm.nih.gov/ (Up to January 1, 2023)	ALL Fields: (erbium, chromium‑doped yttrium, scandium, gallium, and garnet laser OR Er,Cr:YSGG laser OR laser) AND (casein phosphopeptide-amorphous calcium phosphate OR CPP-ACP OR MI paste OR GC Tooth Mousse OR Tooth Mousse) AND (enamel demineralisation OR enamel remineralisation OR white spot lesions OR WSLs OR caries). Sorted by: best match (relevance)
Cochrane Library Cochrane Reviews | Cochrane Library (Up to January 1, 2023)	TITLE, ABSTRACT, KEYWORDS: (erbium, chromium‑doped yttrium, scandium, gallium, and garnet laser OR Er,Cr:YSGG laser OR laser) AND (casein phosphopeptide-amorphous calcium phosphate OR CPP-ACP OR MI paste OR GC Tooth Mousse OR Tooth Mousse) AND (enamel demineralisation OR enamel remineralisation OR white spot lesions OR WSLs OR caries). Sorted by: best match (relevance)
Web of Science http://apps.webofknowledge.com (Up to January 1, 2023)	ALL = (erbium, chromium‑doped yttrium, scandium, gallium, and garnet laser OR Er,Cr:YSGG laser OR laser) AND ALL = (casein phosphopeptide-amorphous calcium phosphate OR CPP-ACP OR MI paste OR GC Tooth Mousse OR Tooth Mousse) AND ALL = (enamel demineralisation OR enamel remineralisation OR white spot lesions OR WSLs OR caries)
EMBASE https://www.embase.com (Up to January 1, 2023)	(erbium, chromium‑doped yttrium, scandium, gallium, and garnet laser OR Er,Cr:YSGG laser OR laser) AND (casein phosphopeptide-amorphous calcium phosphate OR CPP-ACP OR MI paste OR GC Tooth Mousse OR Tooth Mousse) AND (enamel demineralisation OR enamel remineralisation OR white spot lesions OR WSLs OR caries)

### Study selection

Preliminary screening of retrieved studies was performed by browsing titles and abstracts. After removing the duplicated and irrelevant studies, full texts of potential interests were reassessed, and only those meeting inclusion criteria were included. Two reviewers independently accomplished this work. In cases of any disagreements, a third reviewer was consulted and the disagreement was resolved.

### Eligibility criteria

Determine inclusion and exclusion criteria for study selection before initiating a systematic review, based on (PICOS) principles (Table 2).

**Table 2:** Eligibility criteria for the study selection

Category	Inclusion criteria	Exclusion criteria
Participants	• Human teeth • Enamel • No defects, microcracks, caries, restorations, or developmental lesions	• Animal teeth (e.g. bovine teeth) • Not enamel(e.g. root) • Enamel with defects, microcracks, or caries
Intervention	Er,Cr:YSGG laser interacts with the remineralising agents CPP-ACP(e.g. MI paste, Tooth Mousse)	Other remineralising agents(e.g. fluoride, CPP-ACFP) and other lasers
Comparison	Only CPP-ACP	Other remineralising agents
Outcome	The remineralisation efficacy: • Enamel surface microhardness • Demineralised lesion depth	The remineralisation efficacy was not evaluated
Study design	In vitro studies	• Case reports • Reviews • Abstracts • In vivo studies

### Data extraction

An excel data-extraction table was established to summarise the following research characteristics: (1) the name of the first author; (2) the publication date; (3) the type of human tooth; (4) the number of teeth per group; (5) type of product containing CPP-ACP; (6) Er,Cr:YSGG laser wavelength; and (7) outcome report. Two reviewers independently and repeatedly completed this task.

### Assessments of the risk of bias

Two authors independently evaluated the risk of bias in the included studies. Any differences between the reviewers were discussed with a third author until an agreement was reached, if necessary. The risk of bias for the included studies was assessed using the Cochrane Collaboration tool for Systematic Reviews [24]. The assessment item included random sequence generation, allocation concealment, blinding of participants and personnel, blinding of outcome assessment, incomplete outcome data, selective reporting, and other possible sources of bias. Bias in every study was classified as “low risk of bias,” “high risk of bias,” and “unclear risk of bias.” Cochrane Review Manager Version 5.4 was used to generate risks of bias figures.

### Statistical analysis

The statistical analyses were conducted using Review Manager (Version 5.4; Rev Man) and Cochrane Collaboration 2020. The data types of outcome indicators are mainly continuity variables. To avoid the measurement unit inconsistencies caused by the use of different measuring instruments, the standardised mean differences (SMDs) were used with 95% confidence intervals (CIs) to summarise the therapeutic effect of each study. Forest plots were used to illustrate the meta-analysis. Statistical significance was defined as a *p* ≤ 0.05 (Z test), and heterogeneity was assessed with I^2^ [25]. I^2^ ranges from 0 to 100%; values of 25, 50, and 75% represent low, moderate, and high heterogeneity, respectively [26]. The meta-analysis’ fixed or random effects models were determined according to heterogeneity. The fixed effects model was used if all of the included studies showed low heterogeneity. When clinical and methodological heterogeneity were high or I^2^ > 50%, random effects models were used to combine the studies.

## Results

### Literature search

Based on this study’s retrieval strategy, a total of 213 articles were initially obtained via a literature search in the four databases, and 70 duplicates were removed. The titles and abstracts of the remaining 143 records were screened, followed by a full-text assessment of 41 candidate articles. Finally, four trials were included for meta-analysis.

### Characteristics of the included studies

Table 3 shows the details of the included studies. These studies were published from 2014 to 2021. One trial was performed on human premolar teeth [14], one investigation used third molars [27], and two studies were conducted on primary teeth [28, 29]. For products containing CPP-ACP, all studies used GC Tooth Mousse [14, 27–29]. The distribution of laser wavelength is as follows: 2780 nm was chosen in four studies, [14, 27–29]. The effects of remineralisation of enamel were investigated through surface microhardness (SMH) [27–29] and lesion depth [14]. Table 4 shows the parameters of the Er,Cr:YSGG laser used in the included studies in this systematic review.

**Table 3:** Characteristics of the included studies

Author (year)	Type of human tooth	Number of teeth per group	Type of product containing CPP-ACP	Wavelength of Er,Cr:YSGG laser	Outcome report
Adel et al. 2020 [14]	premolars	20	GC Tooth Mousse-GC International Itabashi-Ku, Tokyo, Japan	2780 nm	Lesion depth
Ghelejkhani et al. 2021 [27]	third molars	10	GC Tooth Mousse; GC Corporation, Tokyo, Japan	2780 nm	Vickers hardness number (VHN)
Serdar-Eymirli et al. 2018 [28]	primary molars	15	GC Tooth Mousse, GC Corp, Tokyo, Japan	2780 nm	Vickers hardness number
Subramaniam et al.2014 [29]	primary anterior teeth	10	GC Tooth Mousse‑GC International, Itabashi‑Ku, Tokyo, Japan	2780 nm	Brinell hardness number (BHN)

**Table 4:** Parameters for the Er,Cr:YSGG laser used in the studies

Authors, Year [Reference]	Cooling	Fluence (J/cm^2^)	Output power (W)	Pulse energy (mJ)	Frequency (Hz)	Pulse duration (μs)	Irradiation time (s)
Adel et al. 2020 [14]	Air	8.5	0.25	12.5	20	140	20
Ghelejkhani et al. 2021 [27]	Air and water	8	_	100	10	_	30
Serdar-Eymirli et al. 2018 [28]	Air	_	0.25	_	20	140	10
Subramaniam et al.2014 [29]	Air and water	_	4	_	50	140	20

### Assessments of the risk of bias

**Fig. 2:**
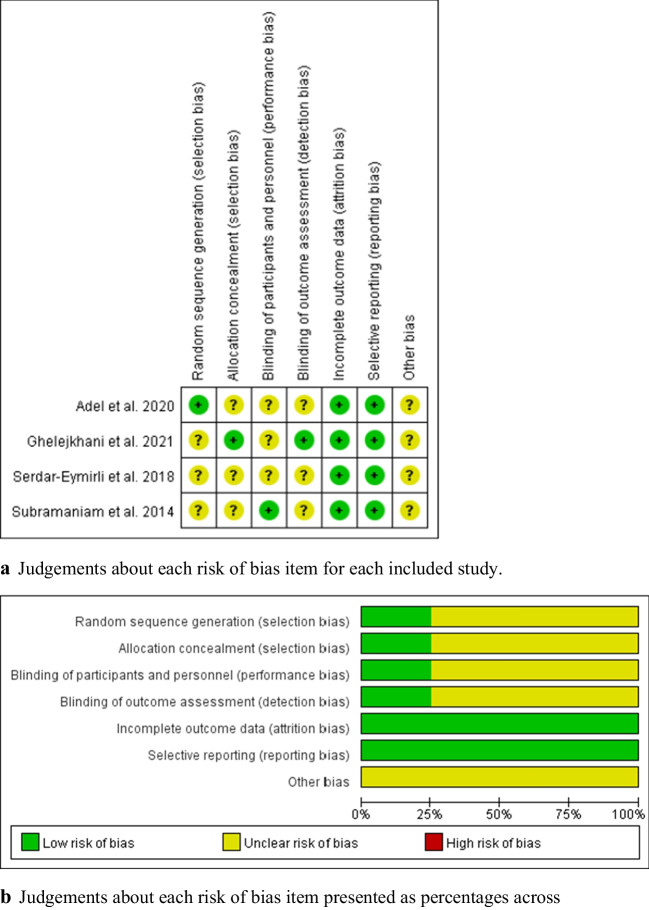
**a** Judgements about each risk of bias item for each included study. **b** Judgements about each risk of bias item presented as percentages across all included studies

Results of the assessment of the risk of bias are shown in Fig. 2a and b. Figure 2a presents an evaluation of each risk of bias item for each included study. Figure 2b shows the judgments about each risk of bias item in this study, presented as percentages across all included studies. Most trials did not report the method of random sequence generation or allocation concealment, and were assessed with an unclear risk of selection bias.

## Data synthesis and meta-analysis

### Surface microhardness

**Fig. 3:**
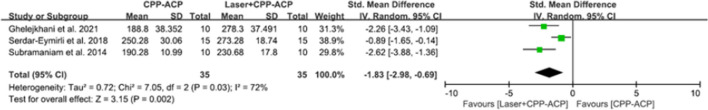
Surface microhardness of CPP-ACP group and laser combined CPP-ACP group

When three studies reporting SMH data were pooled, there was moderate heterogeneity (Tau^2^ = 0.72; Chi^2^ = 7.05, df = 2 [*P* = 0.03]; I^2^ = 72%) [27–29]. The random effects model was chosen. Figure 3 shows the meta-analysis comparing the effect of CPP-ACP + laser combined treatment with CPP-ACP alone on the microhardness of enamel. After analysing three data items, including microhardness, (SMD =  − 1.83, 95% CI: [− 2.98, − 0.69], *P* = 0.002), significant statistical significance indicates that laser + CPP-ACP has a better effect on enamel remineralisation.

### Lesion depth

**Fig. 4:**
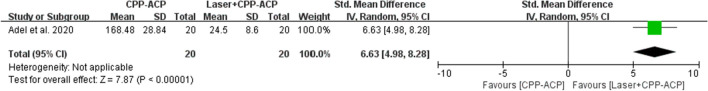
Lesion depth of CPP-ACP group and laser combined CPP-ACP group

Only one study that offers the values of lesion depth was used to assess remineralisation efficacy. The Meta-analysis showed that the combination of laser and CPP-ACP is more advantageous in reducing the lesion depth of demineralisation (SMD = 6.63, 95% CI: [4.98, 8.28], *P* < 0.00001) (Fig. 4).

## Discussion

Demineralisation of the enamel around the fixed orthodontic appliance occurs in WSLs, which is an urgent problem to be solved during and after treatment. The strategy to prevent WSL production can be achieved by inhibiting demineralisation and promoting the remineralisation of early lesions [30]. A systematic review in 2019 pointed out that CPP-ACP has an excellent remineralisation effect in clinical studies and in vitro evaluations [31]. With the increasing popularity of laser application in orthodontics, some scholars have explored the combination of lasers and CPP-ACP to evaluate the effect of remineralisation on early enamel lesions. Recent studies have also shown that lasers can improve the anti-demineralisation ability of tooth enamel [32]. Among various types of lasers, the Er,Cr:YSGG laser has a high absorption coefficient in enamel, so this laser is often used to treat hard tissue diseases of teeth. However, it remains uncertain whether the combination of the Er,Cr:YSGG laser and CPP-ACP will yield superior results. This systematic review summarised evidence from controlled trials and evaluated whether the Er,Cr:YSGG laser enhances the effect of CPP-ACP to promote enamel remineralisation. Finally, out of the initially identified 213 from the literature search in the databases, four trials were included in the quantitative synthesis (meta-analysis).

The microhardness indentation measurement technique can provide qualitative information on enamel mineral changes and obtain the enamel SMH, which has been used to determine demineralisation and remineralisation effects [33]. Three of the studies included in this meta-analysis evaluated enamel surface hardness. Analysis of their combined results shows that the effect of the Er,Cr:YSGG laser combined with CPP-ACP on increasing enamel surface hardness was better than that of CPP-ACP alone, and the difference was statistically significant (*p* = 0.002). This could be attributed to the chemical and morphological changes in tooth enamel caused by laser exposure, enhancing the penetration of the CCP-ACP nanocomplex into the deep layer of the hydroxyapatite crystal [34]; with the increase of calcium and phosphate mineral content in enamel, the SMH value increased. Thereby enhancing the effect of enamel remineralisation.

Polarised light measurements are a highly sensitive technique for showing changes in hard tissues, and they can provide quantitative information on the pore volume (porosity) in demineralised and remineralised enamel and lesion characteristics [33]. The enamel samples were prepared into thin sections and examined under a polarising microscope, which can quantify the depth of demineralised lesions [33]. Among the four studies included, one examined the depth of enamel lesions exposed to acidic conditions using polarised light microscopy to evaluate the effect of the Er,Cr:YSGG laser combined with CPP-ACP in preventing permanent tooth demineralisation. The results show that this combination resulted in significantly less lesion depth compared to the use of CPP-ACP alone.

The above two evaluation indexes suggest that the Er,Cr:YSGG laser can promote the remineralisation effect of CPP-ACP. At the same time, the results of this meta-analysis show high heterogeneous. Due to the limited number of included articles, further subgroup analyses were not performed. The reasons for the high heterogeneity were analysed, and they may be influenced by the following factors.

The SMH value is affected by the laser energy. Within a certain range, the SMH value increases with the increase of laser energy [35]. However, when the energy is higher, larger cracks may occur on the enamel surface, acting as a starting point for acid attack, making the enamel brittle and leading to decreased SMH [15]. In the included studies, the settings of laser parameters were inconsistent, and some studies provided incomplete information on laser parameters, which biased this study’s evaluation of the effect of lasers on enamel demineralisation. In the future, researchers need to provide detailed laser usage parameters to guide clinicians in making correct judgments.

Laser irradiation with or without water mist cooling also plays an essential role in tissue ablation. Studies have shown that the use of a large amount of water during the process of laser irradiation will increase the chance of enamel ablation, as water absorbs part of the energy transmitted by the laser, promoting enamel ablation, and also increasing the porosity of the tooth surface [36, 37]. This could facilitate the diffusion of acids into the enamel structure, increasing the depth of demineralization [38]. However, Hossain and colleagues [39] found that Er:YAG laser irradiation without water mist can sufficiently melt and degenerate enamel, making it highly resistant to demineralisation. More studies are needed to evaluate the effect of laser irradiation with or without water mist on the acid resistance of enamel.

The distance from the laser to the target tissue is also an important influencing factor. In the focused mode, loss of dental tissue can be observed in the irradiated area, even at low energies [40]. Correa-Afonso et al. used an Er:YAG laser with a focusing mode of 12 mm to irradiate the enamel surface, and their results showed that when the irradiation distance was adjusted to 4 mm and accompanied by water cooling, the laser was more effective in preventing enamel demineralization [41].

Although studies have shown that when Er,Cr:YSGG laser is used with subablative parameters and without water cooling, the temperature in the pulp cavity does not rise to the threshold for pulp damages [42, 43]. However, in order to ensure that laser does not damage pulp vitality during clinical use, more studies are needed in the future to evaluate the increase of pulp cavity temperature during the use of the laser and select safe and effective laser parameters for its use.

The complete results of this study may have particular guiding significance for clinical and future research. The combined use of the Er,Cr:YSGG laser and CPP-ACP is superior to using CPP-ACP alone in promoting enamel remineralisation, offering a new option for doctors in future clinical applications. However, further research is needed to investigate the parameter settings of the Er,Cr:YSGG laser and whether water mist cooling is necessary during its use.

## Conclusions

Under the limitations of this meta-analysis, the results show that the Er,Cr:YSGG laser combined with CPP-ACP has a better effect on enamel remineralisation than CPP-ACP alone. The combination of the Er,Cr:YSGG laser and CPP-ACP may be a feasible method to prevent and treat enamel demineralisation.
